# Is age a risk factor for liver disease and metabolic alterations in ataxia Telangiectasia patients?

**DOI:** 10.1186/s13023-017-0689-y

**Published:** 2017-08-04

**Authors:** Talita Lemos Paulino, Marina Neto Rafael, Sonia Hix, David Carlos Shigueoka, Sergio Aron Ajzen, Cristiane Kochi, Fabíola Isabel Suano-Souza, Rosangela da Silva, Beatriz T. Costa-Carvalho, Roseli O. S. Sarni

**Affiliations:** 1 Department of Pediatrics, Escola Paulista de Medicina, Federal University of São Paulo (UNIFESP), Rua dos Otonis, n° 725, Vila Clementino, São Paulo, SP CEP 04025-002 Brazil; 2 Department of Morphology and Physiology, Faculdade de Medicina do ABC, ABC Foundation (FMABC), Santo André, SP Brazil; 3 Department of Diagnostic Imaging, Escola Paulista de Medicina, Federal University of São Paulo (UNIFESP), São Paulo, SP Brazil; 4 Santa Casa de Sao Paulo School of Medical Sciences (FCMSCSP), São Paulo, SP Brazil; 5 School of Nutrition, Federal University of Alfenas (UNIFAL, Alfenas, MG Brazil

**Keywords:** Ataxia Telangiectasia, Atherosclerosis, Carotid Intima-media thickness, Insulin resistance, Fatty liver disease, Dyslipidemia, Nutritional status, Diabetes

## Abstract

**Background:**

Ataxia telangiectasia (A-T) is a neurodegenerative disease that leads to mitochondrial dysfunction and oxidative stress. Insulin resistance (IR), type 2 diabetes and the risk for development of cardiovascular disease was recently associated as an extended phenotype of the disease. We aimed to assess IR; liver involvement; carotid intima-media thickness (cIMT) and metabolic alterations associated to cardiovascular risk in A-T patients, and relate them with age.

**Results:**

Glucose metabolism alterations were found in 54.6% of the patients. Hepatic steatosis was diagnosed in 11/17 (64.7%) A-T patients. AST/ALT ratio > 1 was observed in 10/17 (58.8%). A strong positive correlation was observed between insulin sum concentrations with ALT (*r* = 0.782, *p* < 0.004) and age (*r* = 0.818, *p* = 0.002). Dyslipidemia was observed in 55.5% of the patients. The apolipoprotein (Apo-B)/ApoA-I ratio (*r* = 0.619; *p* < 0.01), LDL/HDL-c (*r* = 0.490; *p* < 0.05) and the Apo-B levels (*r* = 0.545; *p* < 0.05) were positively correlated to cIMT.

**Conclusions:**

Metabolic disorders implicated in cardiovascular and liver diseases are frequently observed in adolescent A-T patients and those tend to get worse as they become older. Therefore, nutritional intervention and the use of drugs may be necessary.

## Background

Clinical and biochemical alterations, such as reduction of lean mass, premature aging, insulin resistance (IR), type 2 diabetes, and risk of developing cardiovascular (CV) disease [1] have been recently added to the classic phenotype of ataxia telangiectasia (A-T).

The disease is caused by mutations in the ataxia-telangiectasia mutated (*ATM*) gene [2] and causes reduction in antioxidant cell capacity and constant oxidative stress that are related to the development of chronic morbidities [3, 4].

ATM-deficient mice showed glucose intolerance, IR, and impaired insulin secretion whose mechanisms are not fully known [5, 6]. A recent study showed high blood glucose and low insulin sensitivity in patients with A-T compared to healthy controls [7].

Literature is still scarce regarding the liver changes observed in A-T patients. A mouse model study emphasized the important role of the ATM pathway in liver fat accumulation and has associated its activation to steatohepatitis-apoptosis and fibrosis – both considered important findings for the progression of nonalcoholic fatty liver disease (NAFLD) [8].

Regarding the CV risk, an ATM study with apolipoprotein (Apo) E-deficient mice has described the emergence of atherosclerotic lesions with accelerated progression in association with IR and glucose intolerance [9]. Furthermore, it was found that ATM deficiency resulted in an increased c-jun N terminal kinase (JNK) activity related to metabolic syndrome [10], failure in the regulation of the Nuclear Factor kappa B (NF-kB) expression, increased production of free radicals, and reduction of oxidative phosphorylation, leading to changes in lipid and glucose metabolism [11].

The CV risk can be assessed by biochemical methods and non-invasive imaging techniques, such as carotid intima-media thickness (cIMT) by ultrasonography (US). A previous study conducted by our group has identified significant changes in triglyceride levels (TG), cholesterol fractions of Non-HDL-c (NHDL-c), and in the relationship between CT/HDL-c and LDL-c/HDL-c in patients with A-T [12].

Given the lack of studies on the metabolic changes observed in A-T involved in the risk of developing chronic diseases, the aim of this study was to assess IR; liver involvement; carotid intima-media thickness (cIMT) and metabolic alterations associated to cardiovascular risk in A-T patients, and relate them with age.

## Methods

In a cross-sectional controlled study, we evaluated 18 A-T patients of both genders, between 5 and 25 years of age, who were diagnosed with A-T according to the criteria of the European Society for Immunodeficiencies (ESID) [13]. The control group was composed by 17 healthy individuals matched in age, gender and pubertal stage; it was used to compare biochemical markers related to cardiovascular risk and food intake. The study was approved by the Research Ethics Committee from the Federal University of São Paulo (UNIFESP). Patients and controls with acute infection at the time of collection were exclude, as well as those using oral corticosteroids or hypoglycemic agents in the 3 months prior to collection.

### Anthropometric evaluation and food intake

The anthropometric evaluation included the measurement of weight, height, mid-upper arm circumference (MUAC) and skinfold thickness (tricipital, subscapular, bicipital, and sacroiliac). The patients who were unable to stand upright were weighed in their parent’s arms and their recumbent height was measured on a firm, flat surface using an inextensible tape that was graduated in millimeters.

In order to classify nutritional status, body mass index to age z-score (ZBMI) for children/adolescents and body mass index (BMI) for adults were calculated. The sum of skinfold thickness and MUAC was used to estimate body composition [14–17]. Pubertal stage was evaluated according to Marshall and Tanner [18].

The assessment of food intake was performed using a 24 h dietary recall (R24hs). The calculation of nutrients in the diet was performed by use of the software Diet Win® and was analyzed according to Dietary Reference Intakes (DRIs) [19]. None of A-T patients had feeding tubes.

### CV risk assessment

The lipid profile [triglycerides (TG), total cholesterol (TC), HDL-c, and LDL-c] was measured with enzymatic-colorimetric tests [20, 21]. The non-HDL cholesterol (NHDL-c) values were obtained by subtracting the HDL-c values from the TC values [21, 22]. Apo A-I, Apo B, small-dense LDL-c particles (sdLDL), oxidized LDL (LDL-ox) and lipoprotein (a) [Lp(a)] were assessed by immune turbid metric assays (ELISAPRO® Human Mabitech Kit).

The assessment of the carotid intima media thickness (cIMT) was performed only in A-T patients in a blinded fashion by a single examiner who used Doppler Ultrasonography (Medison equipment, Accuvix V10 model with linear transducer of high frequency of 6 - 12 MHz). A short length no longer than 0,5 cm of the distal common carotid artery was chosen, at a distance of 1 cm from the bulb, in which three equidistant measurements of cIMT were taken from the far wall and mean values were considered [23].

### Liver involvement

The biochemical markers were collected for hepatic evaluation such as alanine aminotransferase (ALT) and aspartate aminotransferase (AST). AST/ALT ratio > 1 was considered as indicative of liver fibrosis. Hepatic steatosis was evaluated by ultrasonography in a blinded fashion by a single examiner [24]. Liver involvement was considered when the A-T patients presented hepatic steatosis plus ALT higher than 40 U/L (reference value) or only ALT higher than 40 U/L.

### Assessment of IR

Standard 75-g oral glucose tolerance test (OGTT): glucose and insulin levels were measured at 0, 30, 60, 90, and 120 min. Glucose intolerance was considered when, at 120 min, glycaemia was ≥140 mg/dL and <200 mg/dL and IR was considered when the sum of the five insulin levels measured were >300mUI/mL [25].

### Statistical analysis

The statistical package SPSS 24.0 was used for the analysis. Continuous variables were tested for normality. For comparisons between nonparametric variables, the Mann-Whitney or Kruskal-Wallis test was used and, for the parametric variables, the t-Student or ANOVA test was used. The Chi-square test or Fisher’s exact test was used to analyze the association between qualitative variables. We used Pearson’s and Spearman’s correlation coefficient for comparing the analyzed parameters. A significance level of 5% (*p* < 0.05) was adopted.

## Results

The mean age of A-T patients was 13.9 years old, with 15 (83.3%) males and 9 (50%) pre-pubertal. Eleven out of 18 (61.1%) received regular immunoglobulin infusion. The classification of nutritional status by BMI and MUAC are in Table 1 and more than 50% of the patients had dyslipidemia. Glucose metabolism alterations were found in 6/11 (54.6%) patients. One patient was diagnosed with diabetes mellitus (Table 1).

**Table 1:** Characterization of patients with A-T

Variable		N (%)
Age (*n* = 18)	5–15 years	12 (66.6%)
	16–25 years	6 (33.3%)
Nutritional status (*n* = 18)	Malnutrition	6 (33.3%)
	Normal body mass index	11 (61.1%)
	Overweight	1 (5.5%)
Fat mass (*n* = 18)	Low	2 (11.1%)
	Adequate	9 (50.0%)
	High	7 (38.9%)
Mid-upper arm muscle circumference (*n* = 18)	Low	10 (55.5%)
	Adequate	8 (44.4%)
Lipid profile (*n* = 18)	High total cholesterol	10 (55.5%)
	High LDL-c	10 (55.5%)
	High triglyceride	8 (44.4%)
	Low HDL-c	10 (55.5%)
	High NHDL-c	11 (61.1%)
Diabetes mellitus (*n* = 18)	>200 mg/dL	1 (5.5%)
Oral Glucose Tolerance Test (*n* = 11)	Normal	5 (45.4%)
	Glucose intolerance	1 (9.1%)
	Insulin resistance	4 (36.4%)
	Glucose intolerance and Insulin resistance	1 (9.1%)
ALT (*n* = 17)	> 40 U/L	7 (41.2%)
AST/ALT (*n* = 17)	> 1	10 (58.8%)
Hepatic steatosis (*n* = 17)	No	6 (35.3%)
	Mild	7 (41.2%)
	Moderate	4 (23.5%)
Liver involvement (*n* = 17)		7 (41.2%)

N (%)

Legend: *LDL-c* low density lipoprotein cholesterol, *HDL-c* high density lipoprotein cholesterol, *NHDL-c* Non-HDL-c, *ALT* alanine aminotransferase, and *AST/ALT* aspartate aminotransferase/alanine aminotransferase

Hepatic steatosis was diagnosed in 11/17 (64.7%) A-T patients. AST/ALT ratio > 1 was observed in 10/17 (58.8%) (Table 1). The mean ALT and AST levels, and AST/ALT ratio was 37.7 ± 27.1 U/L, 34.3 ± 11.6 U/L e 1.3 ± 0.7 U/L, respectively. It is also important to note those patients with elevated ALT levels, these levels remained elevated at 6-months clinical follow up (data not shown). Table 2 shows individual ALT and AST values for A-T patients.

**Table 2:** Alanine aminotransferase and aspartate aminotransferase values of the A-T group

A-T Group (*n* = 17)	Aspartate aminotransferase (U/L)	Alanine aminotransferase (U/L)
1	25	10
2	21	14
3	35	28.1
4	51	99.8
5	27	12.2
6	24	23.5
7	34	22.3
8	34	18.6
9	66	49
10	19	50
11	27	17
12	36	13
13	30	59
14	41	50
15	40	90.8
16	31	31.8
17	43	52.7

Malnutrition and overweight were observed in 6/18 (33.3%) and 1/18 (5.5%) A-T patients, respectively. In the control group, malnutrition and overweight were verified in 1/17 (5.9%) and 7/17 (41.2%), respectively. Despite the fact, that the mean BMI (17.3 ± 4.0 kg/m^2^ vs 21.3 ± 4.8 kg/m^2^; *p* = 0.010) was lower in A-T patients we observed in this group in comparison to controls higher levels of Apo B (274.1 ± 184.4 mg/mL vs 167.0 ± 46.0 mg/mL; *p* = 0.027); ApoB/Apo A1 ratio (2.1 ± 1.4 vs 1.2 ± 0.3; *p* = 0.018) and Lp(a) (182.8 (31.2;585.8) pg/mL vs 31.2 (31.2;334.9) pg/mL; *p* < 0.001) suggestive ofa more atherogenic lipid profile (Table 3).

**Table 3:** Comparison of the variables in A-T group and control group

Variable		A-T Group (*n* = 18)	Controls (*n* = 17)	*p* Value
Total cholesterol	mg/dL	177.4 ± 33.3	170.0 ± 25.4	0.448^a^
LDL-c	mg/dL	115.4 ± 32.3	107.6 ± 22.3	0.412^a^
HDL-c	mg/dL	43.5 ± 10.8	45.2 ± 8.1	0.596^a^
Triglycerides	mg/dL	94.1 ± 47.2	91.7 ± 45.8	0.880^a^
Non-HDL-cholesterol	mg/dL	134.2 ± 35.7	124.8 ± 25.6	0.377^a^
Total cholesterol/HDL-c	mg/dL	4.3 ± 1.4	3.9 ± 0.9	0.265^a^
LDL/HDL	mg/dL	2.8 ± 1.1	2.5 ± 0.8	0.272^a^
Apo A-I	mg/mL	130.8 ± 27.2	138.8 ± 19.8	0.330^a^
Apo B	mg/mL	274.1 ± 184.4	167.0 ± 46.0	**0.027** ^**a**^
Apo B/Apo AI	mg/mL	2.1 ± 1.4	1.2 ± 0.3	**0.018** ^**a**^
LDL/ApoB	mg/mL	0.56 ± 0.42	0.67 ± 0.15	0.327^a^
Lp(a)	pg/mL	182.8 (31.2;585.8)	31.2 (31.2;334.9)	**0.001** ^**b**^
LDLox	pg/mL	2610.0 ± 985.0	2338.4 ± 982.6	0.533^a^
sdLDL-c	mmol/L	3.3 ± 0.9	3.2 ± 0.9	0.641^a^

^a^Significance level of the t-Student test.^b^ Significance level of the Mann-Whitney test

Legend: *LDL-c* low density lipoprotein cholesterol, *HDL-c* high density lipoprotein cholesterol, *TC* total cholesterol, *Apo AI* apolipoprotein A, *Apo B* apolipoprotein B, *LDLox* oxidized LDL and *Lp(a)* [lipoprotein (a)]

The bold text means the *p*-value of satistical tests (t-Student^1^ or Mann-Witney^2^ are less than *p*< 0,05 (there was statistical significance)

The mean cIMT of A-T patients was 0.42 mm (range: 0.20 and 0.50). The ApoB/ApoA-I ratio (*r* = 0.619; *p* < 0.01), LDL/HDL-c (*r* = 0.490; *p* < 0.05), and the ApoB values (*r* = 0.545; *p* < 0.05) were positively correlated to cIMT.

**Fig. 1:**
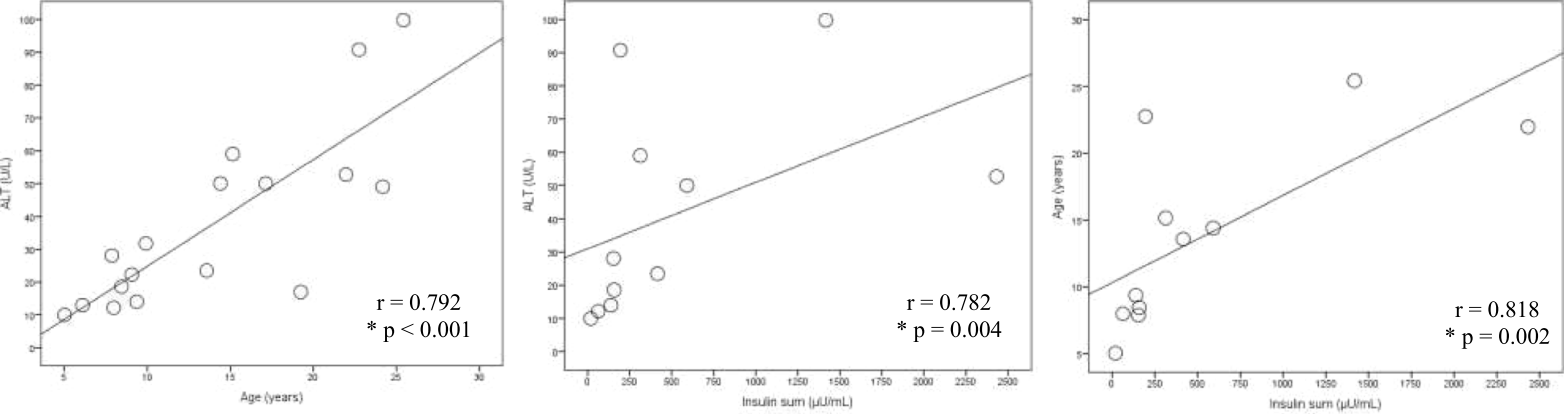
Correlation between insulin sum concentrations of the oral glucose tolerance test (*n* = 11) in patients with A-T with ALT (*n* = 17) and age (*n* = 18). *Significance level of Spearman correlation test

Figure 1 shows the correlation between insulin sum concentrations of the OGTT, in A-T patients, with ALT (*r* = 0.782, *p* < 0.004) and age (*r* = 0.818, *p* = 0.002). We observed a strong positive correlation between insulin sum concentrations with ALT and age. The strongest was with age.

**Fig. 2:**
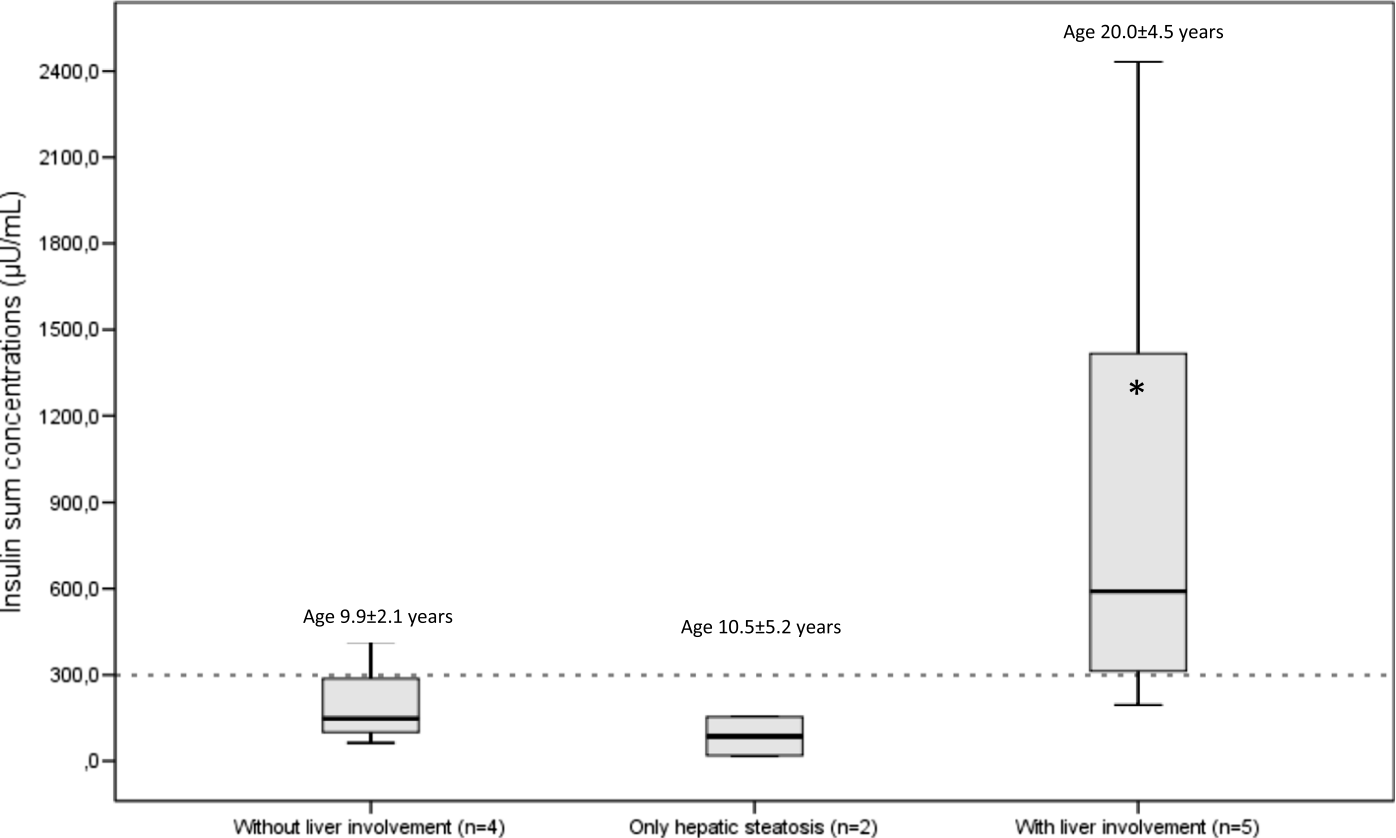
Insulin sum concentrations of the oral glucose tolerance test in A-T patients (*n* = 11) without liver involvement, with hepatic steatosis only and with liver involvement; association with age. * Significance level of the test: ANOVA for patients without liver involvement, only hepatic steatosis and with liver involvement vs age (years), * *p* = 0.001. Kruskal-Wallis test for patients without liver involvement, only hepatic steatosis and with liver involvement vs insulin sum concentrations (μU/mL), * *p* = 0.047

Figure 2 shows that patients who presented with liver involvement had higher sum of insulin levels [590.7 μU/mL (19.1;153.3); *p* = 0.047] and older age (20.2 ± 4.5 years of age; *p* = 0.001) as compared to those patients who had only hepatic steatosis [86.2 μU/mL (19.1;152.3) /10 ± 5.2 years of age] and those without liver involvement [148.1μU/mL (63.1;415.9) /9.9 ± 2.1 years of age].

There were no differences regarding energy and macronutrients intake between A-T group and control group (Table 4).

**Table 4:** Comparison of the means of energy and macronutrients intake in A-T group and control group

Variable		A-T Group (*n* = 18)	Controls (*n* = 17)	*p* Value
Energy	Kcal	1718.4 ± 777.9	1709.5 ± 505.0	0.970^a^
Protein	grams	79.8 ± 35.9	74.4 ± 25.8	0.634^a^
Carbohydrate	grams	237.2 ± 116.5	224.7 ± 81.2	0.729^a^
Total fat	grams	51.2 ± 27.0	57.6 ± 18.4	0.447^a^
Saturated fat	grams	19.6 ± 11.0	21.1 ± 7.8	0.659^a^
Cholesterol	mg	205.9 ± 152.1	189.5 ± 118.7	0.737^a^

^a^Significance level of the t-Student test

## Discussion

This study emphasizes the CV, diabetes, and liver disease risks in A-T patients evidenced by atherogenic lipid profile [higher values of Lp(a) and ApoB/Apo A-I], IR, and presence of hepatic steatosis in 64.7% of the patients. Moreover, it was found that the increase in age was a risk factor for insulin resistance and liver involvement.

The ATM activity seems to be implicated in the glycemic response to metformin in type 2 diabetes [26] and in CV disease [27].

Over time, patients with A-T develop a catabolic condition associated with decline in BMI, chronic lung disease, worsening of hepatic function and glucose metabolism [28], as observed in our study. A recent retrospective cohort study of 55 patients with A-T found endocrine abnormalities, such as diabetes, dyslipidemia, and changes in liver function in two adults [29].

It is known that IR and atherosclerosis are risk factors for developing CV disease, with oxidative stress being related to both complications [30, 31]. IR is also implicated as a key factor in the pathogenesis of steatohepatitis [32], with a positive association of oxidative stress and the severity of liver disease in humans [33]. The deficiency of ATM protein is presented as an important link between the metabolic changes observed in A-T patients.

A recent study, such as observed by us, has found higher glycemia and lower insulin sensitivity in patients with A-T [9]. Some hypotheses can be raised to explain this finding, such as the participation of the ATM in the insulin signaling pathway via phosphorylation of eIF-4E (eukaryotic translation initiation factor 4E) [34] and the regulation exerted by the serine-threonine kinase protein (AKt) or protein kinase B (PKB) activity, which regulates glucose-transporter 4 (GLUT4) translocation by insulin in skeletal muscle and adipose tissue [35].

In our study, abnormalities in glucose metabolism were observed in all pubertal patients who underwent the test. This finding strongly recommends the importance of performing the OGTT in all A-T pubertal patients aimed an early detection of glucose metabolism disorders.

Inflammation is an important factor for the development of obesity-induced IR and it involves tissue immune cells, including phagocytes, lymphocytes, and cytokines [36]. Only one of our patients was obese, showing that this condition is not the cause of IR. Recently, it was demonstrated that the neutrophils from A-T patients produce significantly more cytokines and live longer compared to those from controls, suggesting that innate immune dysfunction may drive inflammation in A-T patients [37]. In a cross-sectional study, the geometric mean of interleukin (IL)-8 level was significantly higher in A-T patients compared with non-A-T (*p* < .0001) [38]. McGrath-Morrow et al. [39] found that approximately 80% of the A-T patients had elevated levels of serumIL-6 and 23.6% having increased levels of IL-6 and IL-8. Furthermore, serum IL-6 levels were correlated with lower lung function.

There are evidences that apolipoproteins are better predictors of CV risk compared to the classic lipid profile [40] and, the Apo B/Apo A-I ratio seems to be a better CV risk predictor [41] that was abnormal in our patients in addiction to the cIMT alteration.

One third of our A-T patients presented undernutrition and 55.5% of them had a compromised body mass. Only 11.1% of the patients had deficit of body fat, which indicates that malnutrition in these patients is associated with reduced lean mass. It is also important to note that of the six patients with glucose metabolism disorders and one with diabetes, five of them had compromised lean mass and six had high NHDL-c values, which reinforces risk factors for developing CV disease dependent on ATM activity (data not shown).

A retrospective cohort study of 53 patients with A-T found liver enzyme abnormalities in 43.4% (23/53) and the presence of steatosis by US in 39% (9/23). Liver biopsy was performed in two patients and showed mild to moderate steatosis in both of them and fibrosis in one of them, supporting our results [42]. Recently, nonalcoholic steatohepatitis without ATM protein in the nucleus of the hepatocytes was showed in a liver biopsy in one A-T patient [43]. One of our patients not included in this study, passed away at 30 years of age with liver cirrhosis, suggesting that this morbidity could affect older A-T patients. The small sample size of this study is a limitation, but for the first time in the literature, we described the association between IR and liver involvement which leads us to recommend the evaluation of glucose metabolism, liver function and US in A-T adolescents. Further studies are necessary to clarify the role of ATM protein in those mechanisms.

## Conclusions

Metabolic disorders implicated in cardiovascular and liver involvement are observed in adolescent A-T patients and those tend to get worse as they become older. Drugs usually employed in diabetes, dyslipidemia and metabolic syndrome have unconvincing results in A-T patients, stressing the need for new treatment alternatives. Therefore nutritional intervention encouraging the use of antioxidants nutrients and new drugs, taking account the pathophysiology of the disease and side effects may be necessary.

## Abbreviations

ALT: Alanine aminotransferase values
ATM: Ataxia telangiectasia mutated
BMI: Body mass index
CIMT: Carotid intima-media thickness
CVD: Cardiovascular disease
DRI: Dietary Reference Intakes
IR: Insulin resistance
JNK: Jun N terminal kinase
MUAC: Mid-upper arm circumference
MUAMC: Mid-upper arm muscle circumference
OGTT: Oral glucose tolerance test
TC: Total cholesterol
TG: Triglyceride levels
US: Ultrassonography
ZBMI: Body mass index to age z-score
